# Nucleoporin 107 is a prognostic biomarker in hepatocellular carcinoma associated with immune infiltration

**DOI:** 10.1002/cam4.5807

**Published:** 2023-03-23

**Authors:** Ju‐sen Nong, Xin Zhou, Jun‐qi Liu, Jian‐zhu Luo, Jia‐mi Huang, Hai‐xiang Xie, Ke‐jian Yang, Jing Wang, Xin‐ping Ye, Tao Peng

**Affiliations:** ^1^ Department of Hepatobiliary Surgery The First Affiliated Hospital of Guangxi Medical University Nanning People's Republic of China; ^2^ Key Laboratory of Early Prevention & Treatment for Regional High Frequency Tumor Ministry of Education, Guangxi Medical University Nanning People's Republic of China

**Keywords:** cell cycle, hepatocellular carcinoma, immune cells, immune checkpoints, nucleoporin 107 (NUP107)

## Abstract

**Objective:**

To assess the diagnostic value and clinical significance of nucleoporin 107 (NUP107) in hepatocellular carcinoma (HCC), and explore the possible mechanisms.

**Methods:**

The transcriptomic and clinical data of HCC patients were retrieved from The Cancer Genome Atlas (TCGA) and GEO databases. Tissue specimens were collected from HCC patients in the Guangxi area. According to the expression levels and prognostic characteristics of NUP107, ROC curves and nomogram models were constructed using the R package.

**Results:**

NUP107 was highly expressed in 26 human cancers including HCC, and was associated with advanced HCC staging and worse prognosis. NUP107 showed satisfactory ability to predict the prognosis of HCC patients (AUC >0.8). Results of gene set enrichment analysis (GSEA) further showed that NUP107 was mainly associated with cell cycle‐related pathways such as the cell cycle, DNA replication, G2M checkpoint, E2F target, and mitotic spindle. In addition, NUP107 was also associated with immune infiltration in HCC and showed significant positive correlation with immune checkpoints (PD‐L1 and TIM‐3).

## Graphical abstract

NUP107 was highly expressed among 26 human cancers including HCC, and associated with advanced HCC staging and worse prognosis. Results of GESA further showed that NUP107 was mainly associated with cell cycle‐related pathways such as the cell cycle, DNA replication, G2M checkpoint, and E2F target along with mitotic spindle. In addition, NUP107 was also associated with immune infiltration in HCC and showed significant positive correlation with immune checkpoints (PD‐L1 and TIM‐3).

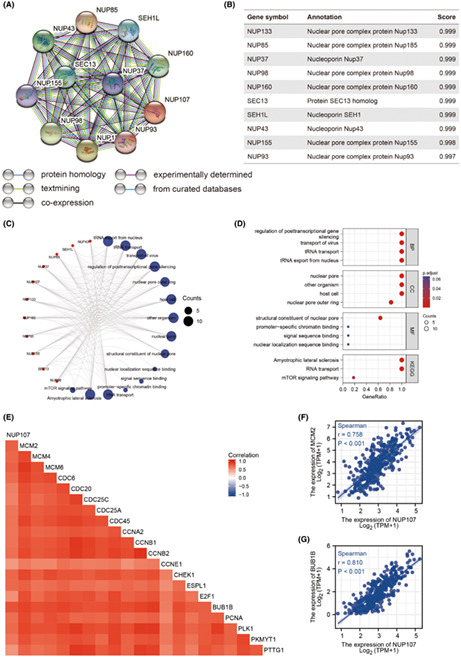

## 1 INTRODUCTION

In 2020, primary liver cancer ranked the sixth globally in terms of incidence, and third in terms of mortality among all malignancies.^1^, ^2^ Despite advances in treatment modalities such as radiofrequency ablation, surgery, liver transplantation, targeted therapy, and immunotherapy, the overall survival (OS) of HCC patients is not satisfactory, mainly due to drug resistance as well as the high rate of postoperative recurrence.^3^ Furthermore, most patients are diagnosed at the advanced stage of the disease,^4^ which precludes the possibility of curative resection. Therefore, novel therapeutic strategies are needed for patients with advanced HCC. The immune microenvironment is critical for tumor progression, invasion, recurrence, and metastasis.^5^, ^6^ Therefore, understanding the role of immune‐related genes can provide new insights into the mechanisms of tumor progression, and help identify novel prognostic biomarkers and drug targets.

In eukaryotic cells, the nuclear pore complex (NPC) is located on the nuclear envelope and acts as a conduit for transport between the cytoplasm and the nucleus.^7^, ^8^ NPCs are assembled from approximately 30 different nucleoporins (NUPS), which are arranged in octagonal symmetry around a central transport channel.^9^, ^10^, ^11^ An NPC consists of three subcomplexes: a loop formed by two NUP107/NUP160 complexes, a core scaffold formed by the NUP93 complex and an inner structure formed by the NUP62 complex.^12^ The NUP107/160 complex is assembled into a Y‐shaped complex consisting of nine NUPS (NUP160, NUP133, NUP107, NUP96/98, NUP85, NUP43, NUP37, SEH1, and SEC13)^13^, ^14^ and plays an important role in the synthesis of NPCs.^15^, ^16^ Studies increasingly show that NPCs are closely related to tumorigenesis.^17^ For example, Sakuma et al. showed that inhibiting NPC formation can selectively induce cancer cell death.^18^ In addition, NUP88 is a novel cancer biomarker that is closely related to tumor progression and invasion, while NUP107 promotes survival of cervical cancer cells.^19^ Furthermore, a recent study showed that NUP160‐SLC43A3 is a recurrent fusion oncogene in patients with angiosarcoma.^20^

NUP107, a key component of the NUP107/160 complex, is located in the core scaffold of NPC, and is a key driver of NPC formation^16^, ^21^ and nucleocytoplasmic molecule trafficking.^22^ During mitosis, NUP107 drives NPC assembly^23^, ^24^ and regulates microtubule polymerization at the kinetochore.^25^ Studies have shown that loss of the NUP107 protein in zebrafish resulted in defective pharyngeal skeleton, intestinal degeneration, and defects in other tissues.^26^ In addition, a strong association has been reported between childhood steroid‐resistant nephrotic syndrome and NUP107 mutations.^27^, ^28^, ^29^ NUP107 exerts a significant effect on cell cycle arrest during DNA damage‐induced genotoxic stress as well,^30^ induces apoptosis,^31^ and regulates the fate of senescent cells through growth factor signaling.^32^ NUP107 is a novel predictive marker of sensitivity to platinum‐based chemotherapy among patients with ovarian cancer.^33^ In addition, NUP107 can also improve the ability of cervical cancer cells to resist oxidative damage.^34^ These findings imply that NUP107 is closely related to tumor genesis and development. The objectives of this study were to elucidate the diagnostic value, prognostic significance and signaling pathways of NUP107 in HCC, and its role in immune infiltration.

## 2 MATERIALS AND METHODS

### 2.1 Patient datasets

Transcriptome sequencing data of 34 human cancers was retrieved from The Cancer Genome Atlas (TCGA) and GTEx through the UCSC XENA platform (https://xenabrowser.net/datapages/).^35^ In addition, the transcriptomic and clinical data of 374 liver cancer tissues and 50 para‐cancerous tissues were obtained from TCGA (https://portal.gdc.cancer.gov/repository). The details are shown in Table 1. The GSE14520 (106 HCC tissues and 106 para‐cancerous tissues), GSE76427 (115 HCC tissues and 52 para‐cancerous tissues), GSE121248 (70 HCC tissues and 37 para‐cancerous tissues), GSE62232 (81 HCC tissues and 11 para‐cancerous tissues), and GSE136247 (39 HCC tissues and 30 para‐cancerous tissues) datasets were downloaded from GEO database (https://www.ncbi.nlm.nih.gov/geo/) for further validation. The details are shown in Table 2. Finally, the amplification and splice mutation data of NUP107 in two HCC datasets (INSERM, Nat Genet 2015, TCGA, Firehose Legacy) were retrieved from cBioPortal for Cancer Genomics website (http://www.cbioportal.org/).

**TABLE 1:** Correlation between nucleoporin 107 (NUP107) expression and clinicopathologic in The Cancer Genome Atlas.

Variables	Patients (*n* = 374)	NUP107 expression		*p*‐value	*χ* ^2^
		Low	High		
*n*		187	187		
Gender					
Female	121	60 (16%)	61 (16.3%)	1.000	0
Male	253	127 (34%)	126 (33.7%)		
Age					
≤60	177	76 (20.4%)	101 (27.1%)	0.011	6.44
>60	196	111 (29.8%)	85 (22.8%)		
T stage					
T1	183	99 (26.7%)	84 (22.6%)	0.360	3.21
T2	95	44 (11.9%)	51 (13.7%)		
T3	80	36 (9.7%)	44 (11.9%)		
T4	13	5 (1.3%)	8 (2.2%)		
Pathologic stage					
Stage I	173	93 (26.6%)	80 (22.9%)	0.129	5.67
Stage II	87	41 (11.7%)	46 (13.1%)		
Stage III	85	35 (10%)	50 (14.3%)		
Stage IV	5	4 (1.1%)	1 (0.3%)		
Histologic grade					
G1	55	40 (10.8%)	15 (4.1%)	< 0.001	21.51
G2	178	94 (25.5%)	84 (22.8%)		
G3	124	46 (12.5%)	78 (21.1%)		
G4	12	4 (1.1%)	8 (2.2%)		
AFP (ng/mL)					
≤400	215	127 (45.4%)	88 (31.4%)	<0.001	16.63
>400	65	19 (6.8%)	46 (16.4%)		
Vascular invasion					
No	208	115 (36.2%)	93 (29.2%)	0.162	1.95
Yes	110	51 (16%)	59 (18.6%)		

**TABLE 2:** Correlation between nucleoporin 107 (NUP107) expression and clinicopathological features in GSE14520.

Variables	Patients (*n* = 212)	NUP107 expression		*p*‐value	*χ* ^2^
		Low	High		
*n*		106	106		
Gender					
F	29	13 (6.1%)	16 (7.5%)	0.689	0.16
M	183	93 (43.9%)	90 (42.5%)		
Age					
>60	37	16 (7.5%)	21 (9.9%)	0.469	0.52
≤60	175	90 (42.5%)	85 (40.1%)		
ALT (>/≤50 U/L)					
High	88	46 (21.7%)	42 (19.8%)	0.676	0.17
Low	124	60 (28.3%)	64 (30.2%)		
Tumor size (>/≤5 cm)					
>5 cm	74	40 (19%)	34 (16.1%)	0.440	0.6
≤5 cm	137	65 (30.8%)	72 (34.1%)		
Multiple/single lobules					
Multiple	45	24 (11.3%)	21 (9.9%)	0.737	0.11
Single	167	82 (38.7%)	85 (40.1%)		
Cirrhosis					
No	17	9 (4.2%)	8 (3.8%)	1.000	0
Yes	195	97 (45.8%)	98 (46.2%)		
TNM stage					
I + II	165	77 (36.3%)	88 (41.5%)	0.098	2.73
III	47	29 (13.7%)	18 (8.5%)		
BCLC stage					
0	20	11 (5.2%)	9 (4.2%)	0.268	3.94
A	143	65 (30.7%)	78 (36.8%)		
B	22	14 (6.6%)	8 (3.8%)		
C	27	16 (7.5%)	11 (5.2%)		
AFP (>/≤300 ng/mL)					
High	94	56 (26.8%)	38 (18.2%)	0.030	4.74
Low	115	50 (23.9%)	65 (31.1%)		

### 2.2 Differential expression analysis

The NUP107 expression levels in 34 human cancers, HCC tumor samples, and adjacent non‐tumor samples were analyzed and compared using Student's *t*‐test. The expression levels of NUP107 in various HCC stages (T stage, pathological stage, histological grade and AFP level) were analyzed with the ggplot2 package using R.

### 2.3 Survival analysis

Survival analysis was performed by the Kaplan–Meier method along with Cox regression model. The Kaplan–Meier method was used to assess the survival of NUP107^high^ and NUP107^low^ patient groups across various clinical subgroups. Cox regression model was used to analyze the effect of multiple factors (age, gender, T staging, pathological staging, histological grading, AFP, and venous invasion) on the survival of HCC patients.

### 2.4 Diagnostic efficiency and nomogram

The diagnostic and predictive power of NUP107 was evaluated by plotting ROC curves using the pROC and timeROC R packages. The area under the curve (AUC) value was calculated, and AUC >0.8 is indicative of satisfactory discriminative ability.^36^ A nomogram was further developed by adding the scores for each prognostic factor. The OS of HCC patients was predicted using the survival and rms R packages. The predictive accuracy of the nomogram was validated through calibration plots.

### 2.5 Screening for differentially expressed gene

The differentially expressed genes (DEGs) between the NUP107^high^ and NUP107^low^ expression HCC samples (cutoff value was 50%) in TCGA database were screened using the DESeq2 R package.^37^ The top 10 DEGs were visualized by heatmaps.

### 2.6 Functional enrichment analysis

The ClusterProfiler package in R was adopted to functionally annotate the DEGs and NUP107‐related genes according to Gene Ontology (GO) terms as well as Kyoto Encyclopedia of Genes and Genomes (KEGG) pathways.^38^

### 2.7 Gene set enrichment analysis

Gene set enrichment analysis (GSEA) was performed using the R package ClusterProfiler (3.14.3) to investigate the differences in biological functions and KEGG pathways between the NUP107^high^ and NUP107^low^ HCC groups (cutoff value was 50%). *p <* 0.05 and false discovery rate (FDR) <0.25 were the criteria for statistical significance.^38^

### 2.8 Immunohistochemistry

Paired tumors and para‐cancerous tissues were collected from the First Affiliated Hospital of Guangxi Medical University from April 2021 to April 2022 from 40 patients with surgically resected and postoperative pathological diagnosis of HCC. The tissue specimens were formaldehyde‐fixed and paraffin‐embedded followed by cutting into sections. According to the instructions of a general two‐step IHC kit (PV‐9000, ZSGB‐Bio, China), the tissue sections were incubated overnight with anti‐NUP107 antibody (1:400, Proteintech) at 4°C, and thereafter with the enhanced enzyme‐labeled goat anti‐mouse/rabbit IgG polymer for 30 min. Finally, the tissue sections were stained with DAB chromogenic kit (ZLI‐9018, ZSGB‐Bio, China) and counterstained with hematoxylin. Two pathologists independently examined and scored the tissues.

### 2.9 Immune infiltration analysis

TIMER (https://cistrome.shinyapps.io/timer/) is a web server used for analyzing the correlation between immune cells and gene expression levels in the TCGA dataset.^39^, ^40^ The website TIMER and GSVA package in R were used to evaluate the expression level of NUP107 in different immune cells and its correlation with the degree of immune infiltration. *p <* 0.05 and correlation coefficient >0.300 were the criteria for statistical significance.

### 2.10 Statistical analysis

Statistical analysis was performed using R studio (Version 1.2.5033, R 3.6.2). NUP107 expression between tumor and para‐tumor tissues were compared by Student's *t*‐test. Survival analysis was performed using the Kaplan–Meier method and Cox regression model. Multivariate COX regression analysis was performed to screen for the prognostic factors. Diagnostic value was assessed by ROC analysis. Spearman's correlation test was performed to analyze the correlation between two groups. *p <* 0.05 was considered statistically significant.

## 3 RESULTS

### 3.1 NUP107 is highly expressed in human cancers and HCC

**FIGURE 1:**
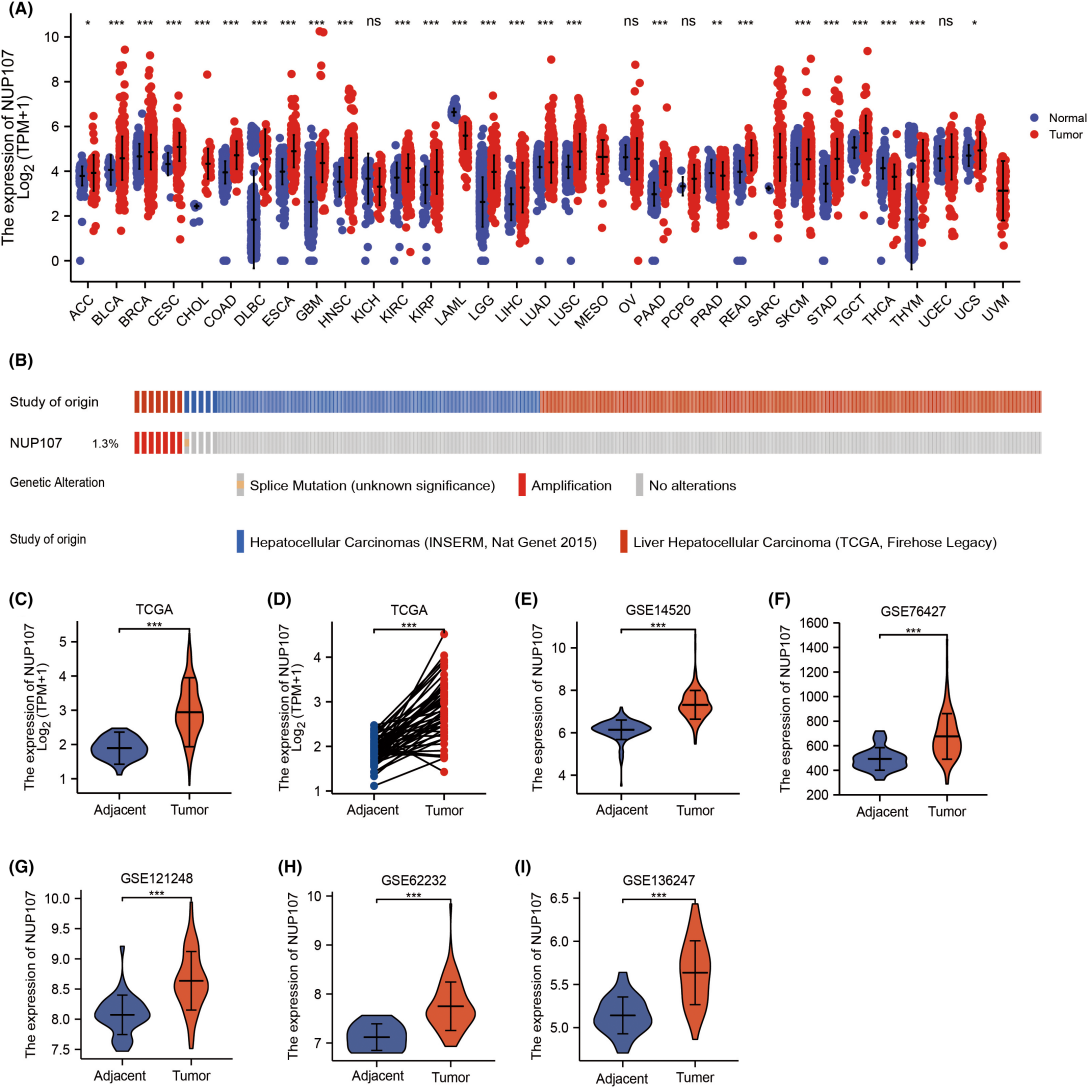
Nucleoporin 107 (NUP107) is highly expressed in human cancers and hepatocellular carcinoma (HCC). (A) Expression levels of NUP107 in 31 different human cancers, ns: *p* ≥ 0.05; *p < 0.05; **p < 0.01; ***p < 0.001. (B) NUP107 expression profile in the cBioPortal OncoPrint plot. The expression levels of NUP107 in (C) 370 HCC tissues and 50 para‐carcinoma tissues in The Cancer Genome Atlas (TCGA) LIHC dataset, (D) 50 paired HCC tissues and para‐carcinoma tissues in TCGA LIHC dataset, (E) 106 HCC tissues and 106 para‐carcinoma tissues in GSE14520 dataset, (F) 115 HCC tissues and 52 para‐carcinoma tissues in GSE76427 dataset, (G) 70 HCC tissues and 37 para‐carcinoma tissues in GSE121248 dataset, (H) 81 HCC tissues and 11 para‐carcinoma tissues in GSE62232 dataset, (I) 39 HCC tissues and 30 para‐carcinoma tissues in GSE136247 dataset.

We analyzed NUP107 mRNA levels across 33 human cancers and the corresponding normal tissues, and found that NUP107 was significantly elevated in 26 cancer types, such as liver, bladder, colorectal, breast, prostate, lung, and thyroid cancers. However, no significant difference was observed in NUP107 expression between the tumor and normal tissues in renal clear cell carcinoma, ovarian serous, pheochromocytoma, paraganglioma, and uterine corpus endometrioid carcinoma (Figure 1A). We also analyzed the genome and copy number of NUP107 in two HCC datasets in the cBioPortal for Cancer Genomics website (INSERM, Nat Genet 2015, TCGA, Firehose Legacy), and found that the frequency of NUP107 gene amplification and splice mutation was 1.3% (Figure 1B). NUP107 expression was also significantly higher in the HCC tissues compared to the paired/unpaired normal liver tissues in TCGA, GSE14520, GSE76427, GSE121248, GSE62232, and GSE136247 datasets (Figure 1C–I).

### 3.2 High NUP107 expression portends poor prognosis in patients with HCC

**FIGURE 2:**
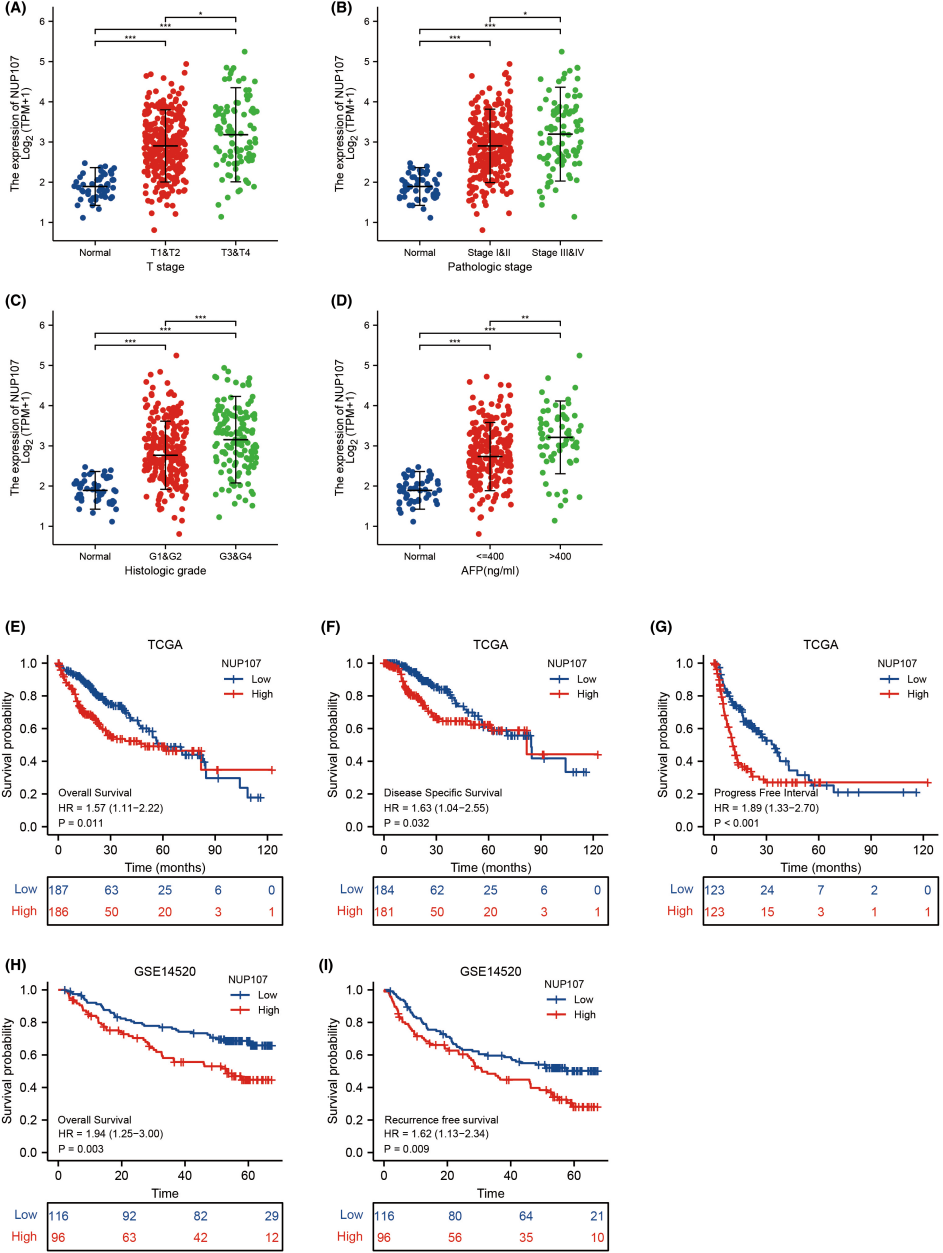
High expression of nucleoporin 107 (NUP107) was associated with more advanced hepatocellular carcinoma staging and poor prognosis. Dot plots show the expression levels of NUP107 in different T‐stages (A), pathological stages (B), histological grades (C), and AFP levels (D). Kaplan–Meier curves showing (E) overall survival (OS), (F) disease‐specific survival and (G) PFS in The Cancer Genome Atlas (TCGA), and (H) OS and (I) relapse‐free survival in GSE14520, * *p <* 0.05; ** *p <* 0.01; *** *p <* 0.001.

**FIGURE 3:**
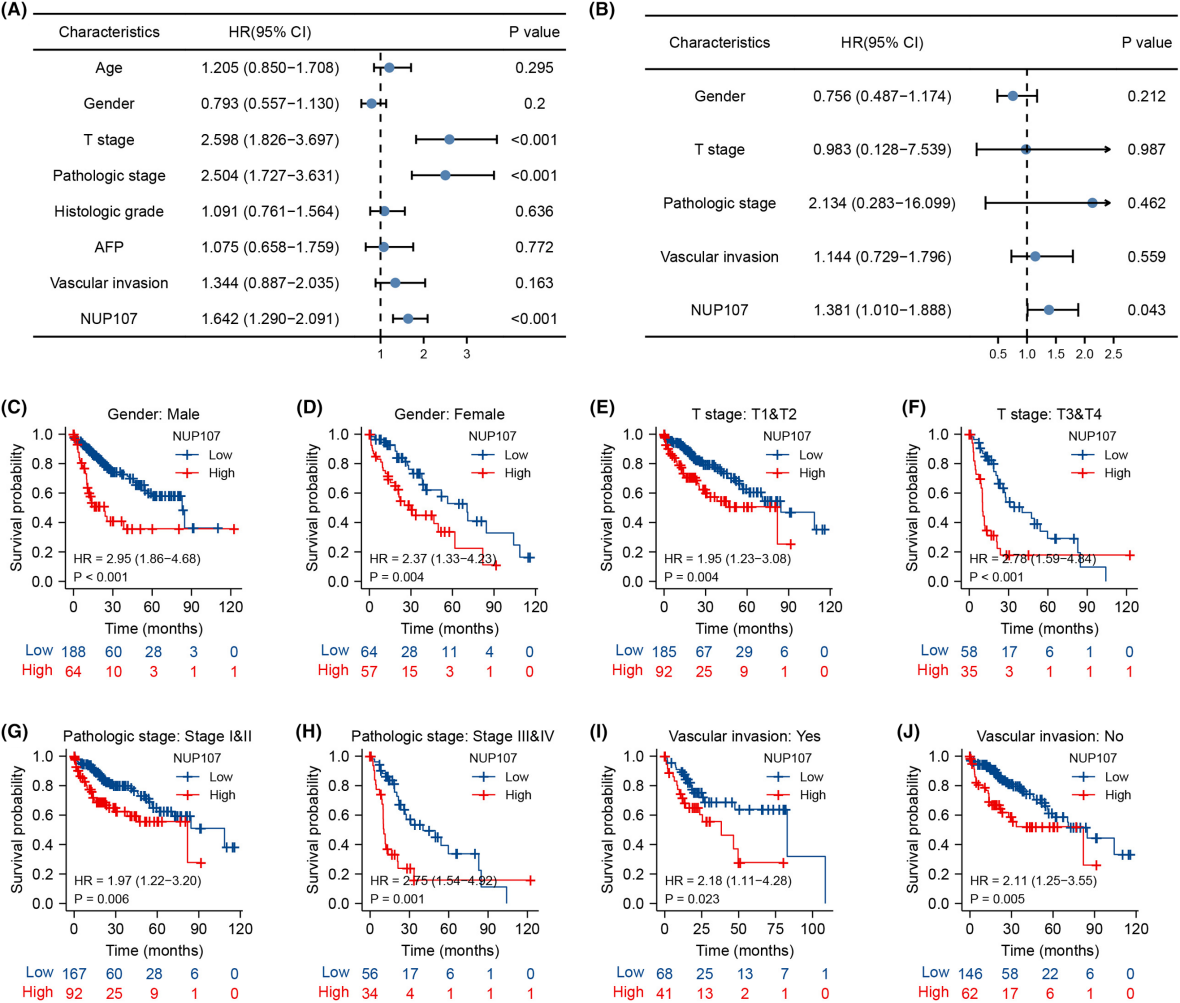
Prognostic value of nucleoporin 107 (NUP107) in hepatocellular carcinoma (HCC). (A) Univariate Cox regression model. (B) Multivariate Cox regression model. Kaplan–Meier survival curves of NUP107^high^ and NUP107^low^ patients in (C) male, (D) female, (E) T1 and T2, (F) T3 and T4, (G) stage I and II, (H) stage III and IV, (I) vascular invasion, and (J) non‐vascular invasion subgroups.

To assess the prognostic relevance of NUP107 in patients with HCC, we analyzed its expression levels across different HCC stages (T‐stage, pathological stage, and histological grading) and AFP levels. NUP107 overexpression was related to more advanced HCC stages (Figure 2A–C), as well as higher AFP levels (Figure 2D). In addition, NUP107 overexpression was associated with worse OS (*p* = 0.011), disease‐specific survival (DSS) (*p* = 0.032), and progression‐free interval (*p <* 0.001) in TCGA (Figure 2E–G). In the GSE14520 dataset, NUP107 overexpression correlated with worse OS (*p* = 0.003) and relapse‐free survival (RFS) (*p* = 0.009) (Figure 2H,I). Univariate analysis further showed that NUP107 (high versus low, HR 1.624, *p <* 0.001), T staging (T1 and T2 versus T3 and T4, HR 2.598, *p <* 0.001), and pathological staging (stages I and II versus III and IV, HR 2.504, *p <* 0.001) were significantly associated with poor prognosis (Figure 3A). Multivariate analysis demonstrated that NUP107 overexpression was an independent risk factor for HCC (*p <* 0.05) (Figure 3B), especially in the male (*p <* 0.001), female (*p* = 0.004), T1 and T2 (*p* = 0.004), T3 and T4 (*p <* 0.001), stage I/II (*p* = 0.006), stage III/IV (*p* = 0.001), vascular invasion (*p* = 0.023), and no vascular invasion (*p* = 0.005) subgroups (Figure 3C–J). Taken together, NUP107 overexpression is a risk factor for HCC.

### 3.3 Diagnostic and predictive ability of NUP107 in HCC

**FIGURE 4:**
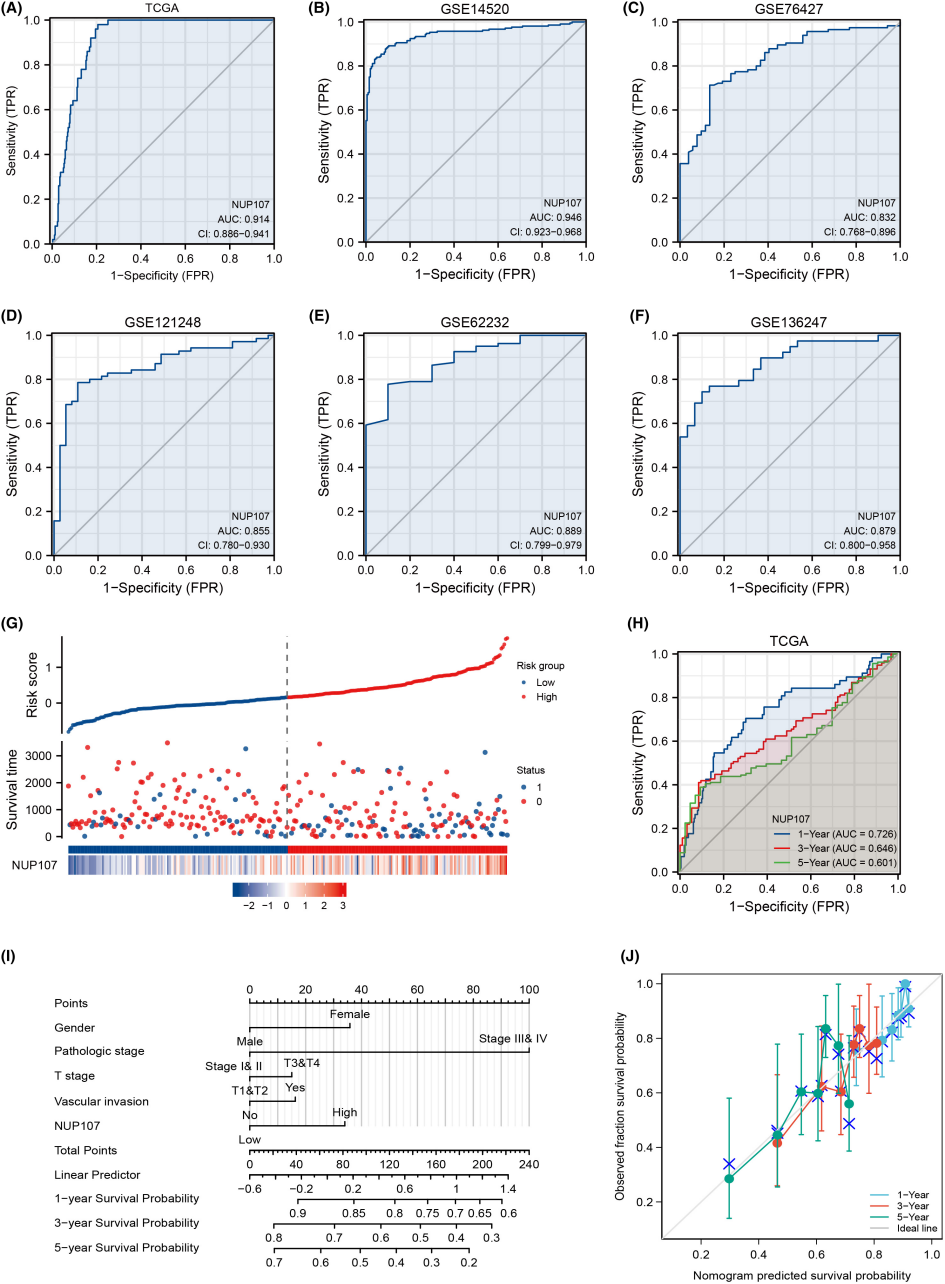
Predictive ability of nucleoporin 107 (NUP107) for hepatocellular carcinoma (HCC). (A–F) Diagnostic ROC curves in The Cancer Genome Atlas (TCGA), GSE14520, GSE76427, GSE121248, GSE62232, and GSE136247. (G) Scatter plot of survival status and NUP107 expression heat map corresponding to HCC patients sorted by risk score. (H) Predictive power of NUP107 for 1‐, 3‐, and 5‐year overall survival (OS) in HCC patients by time‐dependent ROC analysis. (I) Prediction of 1‐, 3‐, and 5‐year OS by column line plots. (J) Calibration plots were used to validate the column line graph model.

The diagnostic and predictive utility of NUP107 for HCC was evaluated by ROC analysis. NUP107 exhibited satisfactory diagnostic ability (AUC >0.8) in several datasets, including TCGA (AUC = 0.914, Figure 4A), GSE14520 (AUC = 0.946, Figure 4B), GSE76427 (AUC = 0.832, Figure 4C), GSE121248 (AUC = 0.855, Figure 4D), in GSE62232 (AUC = 0.889, Figure 4E), and GSE136247 (AUC = 0.879, Figure 4F). In addition, the time‐dependent ROC curves constructed using TCGA data also indicated good predictive ability, with AUC values above 0.6 for 1‐, 3‐, and 5‐year OS (Figure 4H). More patients in the high‐risk score group reached the end event in a shorter duration (Figure 4G). To further predict 1‐, 3‐, and 5‐year survival in patients with HCC, we developed a nomogram on the basis of gender, T‐stage, pathological stage, vascular invasion, and NUP107 expression level (Figure 4I). We obtained the total score by adding the scores for each prognostic factor, and the OS of HCC patients was predicted by determining the probability of the endpoint event by the total score corresponding to the outcome axis. NUP107 expression level was a better predictor of prognosis compared to T‐stage or vascular invasion. The calibration plots of the prediction model suggested that the predicted outcome was less biased (Figure 4J).

**FIGURE 5:**
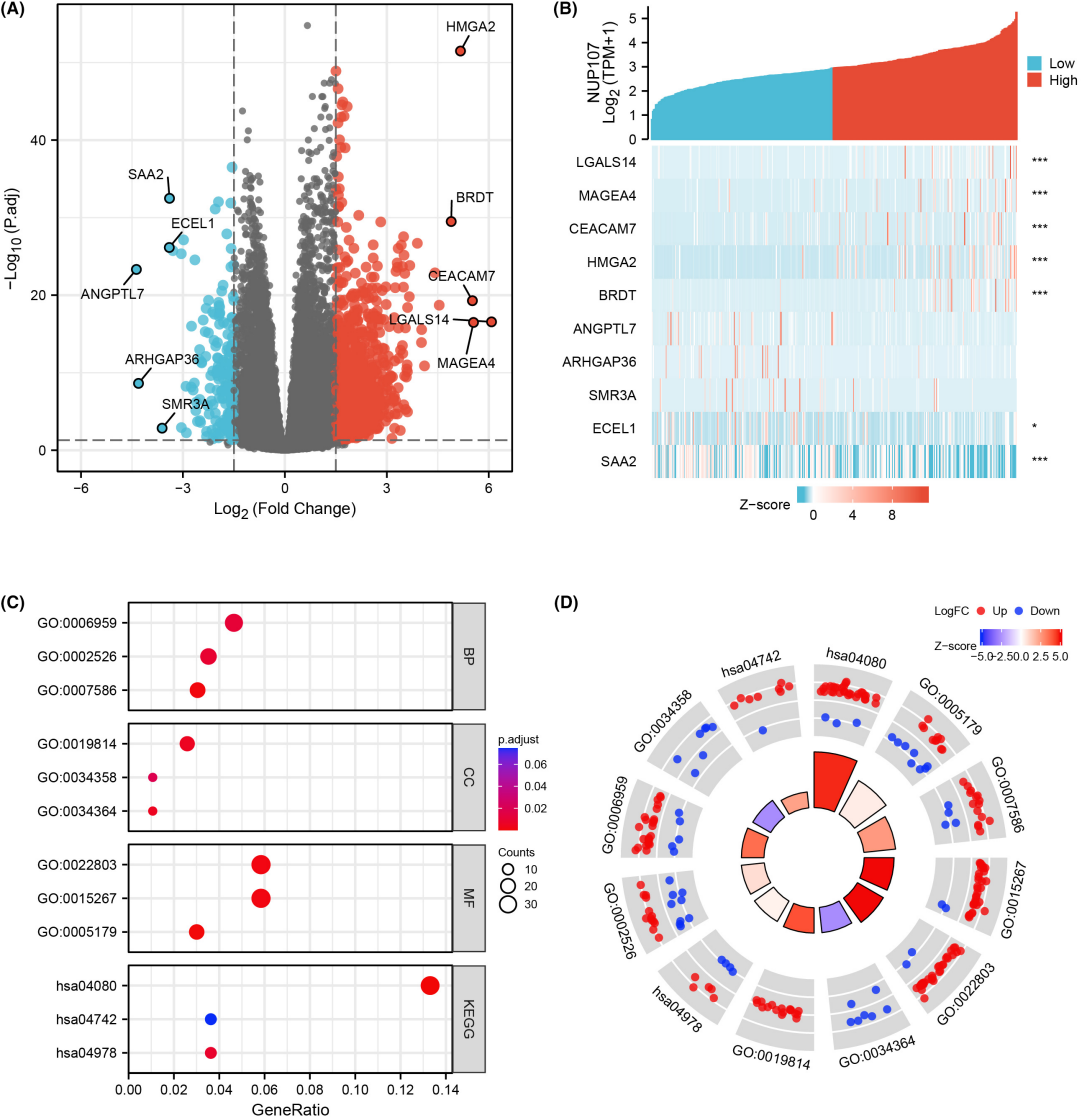
Identification and functional annotation of differentially expressed gene (DEGs). (A) Volcano plot showing the DEGs between NUP107^high^ and NUP107^low^ groups. (B) Heat map showing the top five upregulated and downregulated genes. (C) Bubble plot of the significantly enriched Gene Ontology (GO) terms and Kyoto Encyclopedia of Genes and Genomes (KEGG) pathways. (D) Circle diagram showing the GO and KEGG terms corresponding to the DEGs.

### 3.4 Validation of NUP107 using patient samples

**FIGURE 6:**
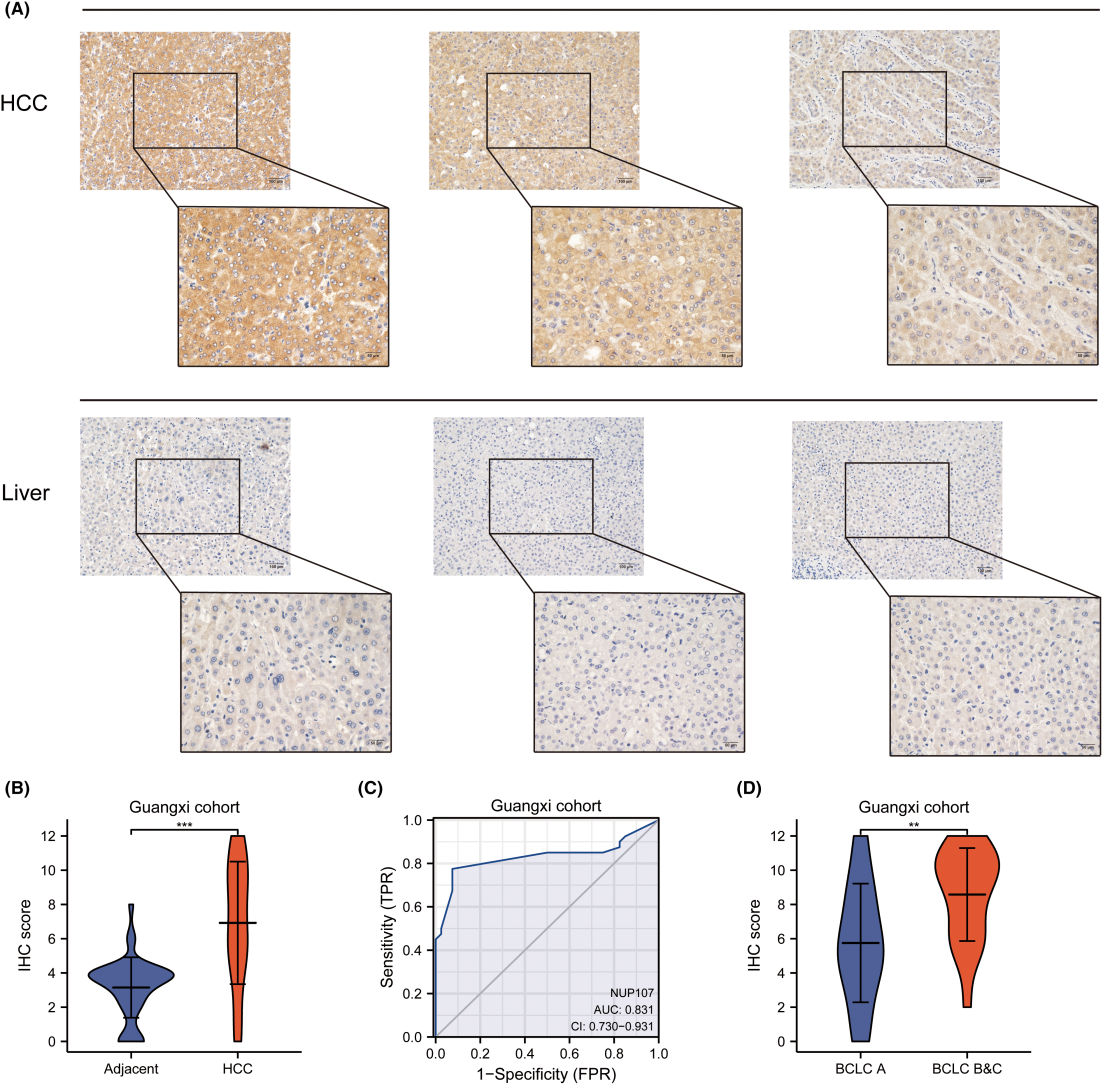
Validation of nucleoporin 107 (NUP107) in liver cancer patients in Guangxi. (A) Representative immunohistochemistry (IHC) images showing in situ expression of NUP107 in hepatocellular carcinoma (HCC) and adjacent liver tissues. (B) Violin chart showing the immunostaining scores of NUP107 in HCC and adjacent liver tissues in the Guangxi cohort. (C) Diagnostic ROC curve of NUP107 for the Guangxi cohort. (D) Expression level of NUP107 in the different BCLC stages.

The results obtained so far were further validated on 40 paired HCC and para‐tumor tissues collected from the First Affiliated Hospital of Guangxi Medical University. Compared to the adjacent tissues, the in situ expression of NUP107 in HCC tissues was stronger (Figure 6A), which corresponded to significantly higher IHC scores in the latter (*p <* 0.001, Figure 6B). Furthermore, NUP107 showed satisfactory diagnostic performance in the Guangxi cohort (AUC = 0.831, Figure 6C). We also verified that NUP107 overexpression was associated with BCLC staging of more advanced HCC in this cohort (*p <* 0.01, Figure 6D).

### 3.5 DEG identification and functional enrichment analysis

We identified 1260 DEGs between the NUP107^high^ and NUP107^low^ HCC samples in TCGA cohort, including 1080 upregulated and 180 downregulated genes (Figure 5A). The top 5 upregulated/downregulated genes are shown in the heat map in Figure 5B. The DEGs were functionally annotated by GO and KEGG functional enrichment analyses. The significantly enriched biological process (BP) consisted of humoral immune response, acute inflammatory response and digestion, the cellular components (CC) included HDL particles, immunoglobulin complexes and plasma lipoprotein particles, and the significant molecular functions (MF) were passive transmembrane transporter protein activity, channel activity, and hormonal activity (Figure 5C,D). The prominent KEGG pathways associated with the DEGs included neuroactive ligand–receptor interactions, mineral uptake and taste transduction.

### 3.6 Results of GSEA

**FIGURE 7:**
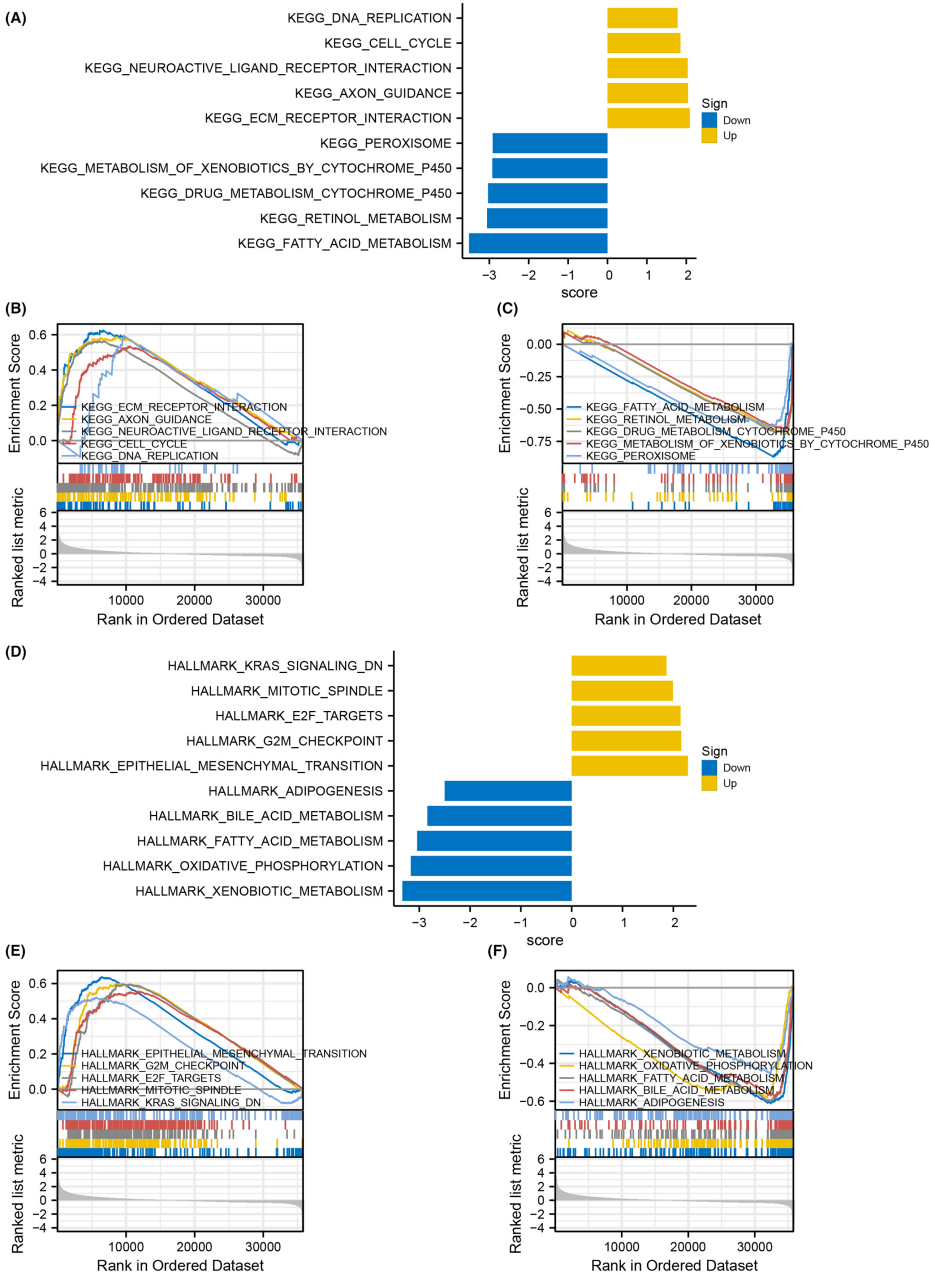
Enrichment plots and normalized enrichment score (NES) visualization of gene set enrichment analysis. (A) Bar graph showing the NES values of the top five Kyoto Encyclopedia of Genes and Genomes (KEGG) pathways positively and negatively correlated with nucleoporin 107 (NUP107). Enrichment plots showing the top five (B) positively and (C) negatively correlated KEGG pathways. (D) Bar graphs showing the NES values of the top five HALLMARK pathways positively and negatively correlated with NUP107. Enrichment plots showing the top five (E) positively and (F) negatively associated HALLMARK pathways.

To further understand the BPs associated with NUP107 and identify the key signaling pathways involved in HCC, we performed GSEA between the NUP107 expression groups. There were several pathways in the Molecular Signature Database (MSigDB) (C2.cp.v7.2.symbols.gmt and h.all.v7.2.symbols.gmt) that showed significant differences between the NUP107^high^ and NUP107^low^ groups (FDR <0.05, ADJ *p <* 0.05). We ranked the signaling pathways on the basis of the normalized enrichment score and obtained the signaling pathways that were most significantly enriched (Figure 7A,D). The five KEGG‐annotated pathways positively associated with high NUP107 expression were ECM receptor interaction, axon guidance, neuroactive ligand–receptor interaction, cell cycle and DNA replication (Figure 7B), and those with a positive association with low NUP107 expression were fatty acid metabolism, retinol metabolism, drug metabolism cytochrome P450, metabolism of xenobiotics by cytochrome P450 and peroxisome (Figure 7C). As shown in Figure 7E, the top five HALLMARK‐related annotations associated with high NUP107 expression were G2/M checkpoint, epithelial mesenchymal transition, E2F target, mitotic spindle, and inhibition of KRAS signaling. Furthermore, adipogenesis, fatty acid, bile acid and xenobiotic metabolism, as well as oxidative phosphorylation were the top 5 HALLMARK terms positively correlated to low NUP107 expression (Figure 7F). These results indicate that overexpression of NUP107 may interfere with cell cycle and mitosis in HCC.

**FIGURE 8:**
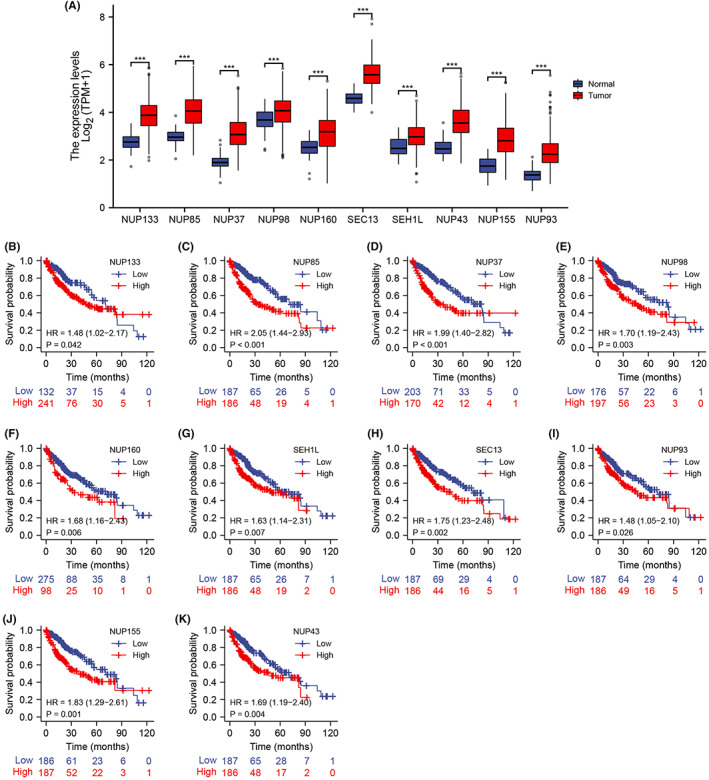
(A) Expression levels of 10 functional partners of nucleoporin 107 in hepatocellular carcinoma (HCC) and para‐carcinoma tissues. Survival curves of HCC patients in The Cancer Genome Atlas cohort demarcated in the basis of (B) NUP133, (C) NUP85, (D) NUP37, (E) NUP98, (F) NUP160, (G) SHE1L, (H) SEC13, (I) NUP93, (J) NUP155, and (K) NUP43 expression. * *p <* 0.05; ** *p <* 0.01; *** *p <* 0.001.

### 3.7 NUP107 and its related genes are strongly correlated with cell cycle genes in HCC

**FIGURE 9:**
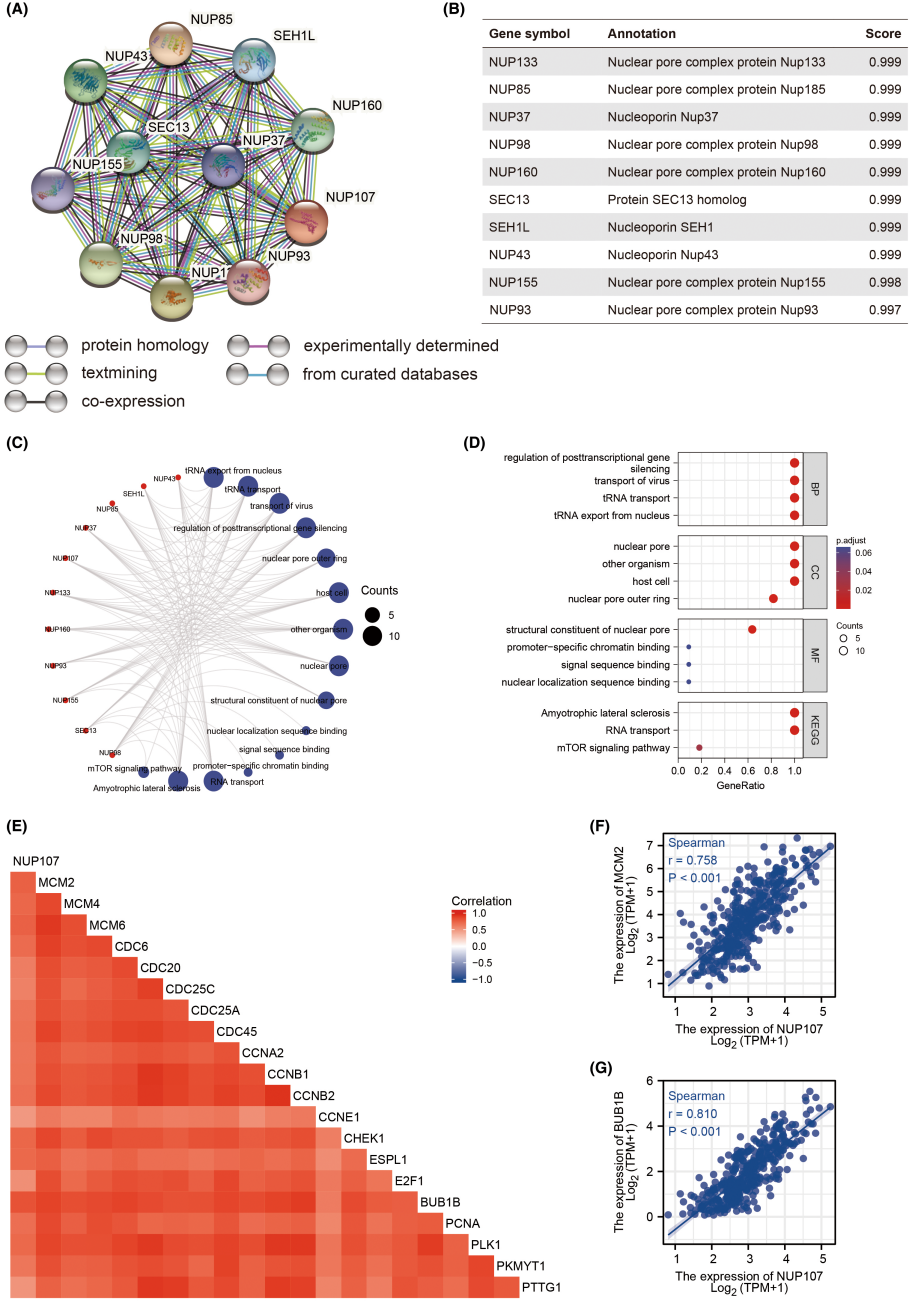
Nucleoporin 107 (NUP107) and its related genes are strongly correlated with cell cycle genes in HCC. (A) PPI network of NUP107‐related genes. (B) Annotation and correlation coefficients of 10 NUP107‐related genes. (C, D) Enriched GO terms and KEGG pathways for the NUP107‐related genes. (E) Correlation matrix showing the correlation between NUP107 and cell cycle regulatory genes including (F) BUB1B and (G) MCM2.

A protein–protein interaction network of the NUP107‐related genes was constructed through the STRING website. NUP133, NUP85, NUP37, NUP96/98, NUP160, SEC13, SEH1L, NUP43, NUP155, and NUP93 were identified as the 10 genes that interacted most with NUP107 (Figure 9A,B). Eight of these 10 functional partners have also been identified by Lutzmann et al.^14^ In addition, NUP133, NUP160, NUP37, NUP107, NUP96/98, NUP43, SEH1, NUP85, and SEC13 are part of the NUP107/160 complex. According to GO analysis, the most significantly enriched BP terms among the NUP107‐related genes were post‐transcriptional gene silencing, viral translocation, tRNA translocation and regulation of tRNA export from the nucleus, the main CC terms included nuclear pore, other organisms, host cells and nuclear pore outer ring, and the MF terms were structural components of the nuclear pore, promoter‐specific chromatin binding, signal sequence binding, and nuclear localization sequence binding. Furthermore, amyotrophic lateral sclerosis, RNA translocation, and mTOR signaling pathway were the KEGG pathways that were most significantly enriched (Figure 9C,D). The above results suggested that NUP107 may influence the progression of HCC via regulation of the cell cycle.

Based on the results of GO and KEGG functional enrichment analysis, we assessed the relationship of NUP107 with cell cycle regulatory genes involved in HCC progression.^41^ As shown in Figure 9E, BUB1B, CDC6, CDC20, CDC25A, CDC25C, CDC45, PLK1, MCM6, CCNB1, CCNB2, CHEK1, ESPL1, E2F1, MCM4, PTTG1, PCNA, CCNE1, PKMYT1, MCM2, and CCNA2 were strongly correlated (*r* > 0.5, *p <* 0.001) with NUP107, especially BUB1B (*r* = 0.810, *p <* 0.001) and MCM2^42^ (*r* = 0.758, *p <* 0.001) (Figure 9F,G). Thus, NUP107 plays a role in the regulation of the HCC cell cycle.

The expression of these genes in TCGA database was further analyzed, which showed that all 10 genes were significantly upregulated in HCC tissues compared to the para‐tumor tissues (*p <* 0.001; Figure 8A). Furthermore, NUP160 (*p* = 0.006), NUP133 (*p* = 0.042), NUP98 (*p* = 0.003), NUP85 (*p <* 0.001) NUP43 (*p* = 0.004), NUP37 (*p <* 0.001), SEH1 (*p* = 0.007), SEC13 (*p* = 0.002), NUP155 (*p* = 0.001), and NUP93 (*p* = 0.026) were significantly associated with the OS of HCC patients (Figure 8B–K). Taken together, NUP107 and other NUP107/160 complex‐related genes are prognostic indicators for HCC.

### 3.8 Role of NUP107 in immune infiltration

**FIGURE 10:**
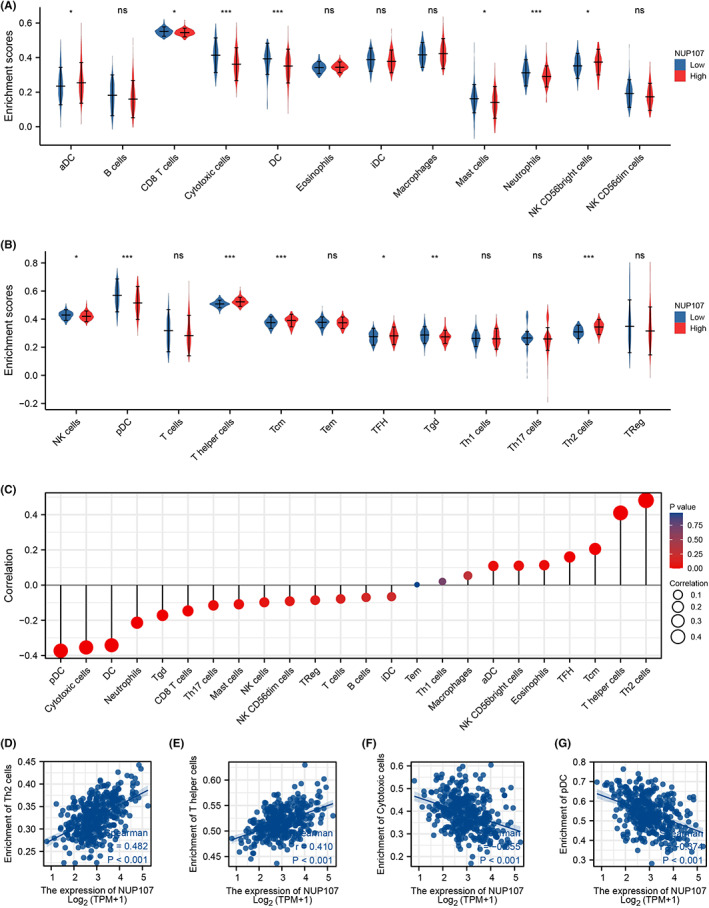
Correlation between nucleoporin 107 (NUP107) expression and immune infiltration in hepatocellular carcinoma. (A, B) Violin plots showing the degree of infiltration of different immune cells in the NUP107^high^ and NUP107^low^ groups. (C) Bubble plots showing the correlation between NUP107 and different immune cells. (D–G) Scatter plots showing the correlation between NUP107 expression levels and (D) Th2 cells, (E) T helper cells, (F) cytotoxic T cells, and (G) plasma DCs. ns: *p ≥* 0.05; * *p <* 0.05; ** *p <* 0.01; *** *p <* 0.001.

HCC is closely related to immune responses during its onset, growth, metastasis, and treatment.^43^ The relationship between NUP107 expression and immune infiltration in HCC was explored through GSVA package in R. We observed a higher infiltration of CD8+ T cells, mast cells, cytotoxic cells, dendritic cells (DCs), plasmacytoid dendritic cells (pDCs), neutrophils, NK cells, and γδ T cells in the NUP107^low^ group, whereas NUP107 overexpression was associated with higher infiltration of NK CD56^bright^ cells, NK cells, T helper cells, central memory T cells, follicular helper T cells, and Th2 cells (Figure 10A,B). Furthermore, NUP107 expression correlated positively with the infiltration of Th2 cells (*r* = 0.482, *p <* 0.001; Figure 10D), T helper cells (*r* = 0.410, *p <* 0.001; Figure 10E), central memory T cells, follicular helper T cells, eosinophils, NK CD56^bright^ cells as well as activated DCs (Figure 10C), but negatively with cytotoxic T cells (*r* = −0.355, *p <* 0.001; Figure 10F), pDCs (*r* = −0.374, *p <* 0.001; Figure 10G), immature DCs, B cells, T cells, Tregs, NK CD56^dim^ cells, NK cells, mast cells, Th17 cells, CD8 T cells, γδ T cells, neutrophils, and DCs (Figure 10C). The Th1/Th2 ratio is skewed in liver, lung, and breast tumors,^44^, ^45^, ^46^ and the predominance of the Th2 subtype^47^ and T helper cells in general are associated with immune escape of tumor cells.^48^ Therefore, our findings suggest that NUP107 may promote tumor growth by increasing the infiltration of Th2 cells, central memory T cells, follicular helper T cells, T helper cells and other immunosuppressive populations, and decreasing that of cytotoxic T cells, pDCs, immature DCs, B cells, T cells, Tregs, and NK cells.

Chemokines along with their receptors exert a pivotal effect on the anti‐tumor immune response.^49^ We found that NUP107 was positively correlated with several chemokines and the specific receptors (Figure S2A,S2D), including CCL28 (*r* = 0.377, *p* < 0.001), CXCL8 (*r* = 0.310, *p* < 0.001), CCR8 (*r* = 0.455, *p* < 0.001), and CXCR4 (*r* = 0.392, *p* < 0.001) (FigureS2B, S2C, S2E, S2F). Immune checkpoint inhibitors (ICIs) are able to prolong the survival of patients with cancers, and the response to ICIs relies on the expression levels of immune checkpoint genes.^50^ Therefore, we next examined the correlation between NUP107 and HCC‐associated immune checkpoint genes, including PD‐1, PD‐L1, PD‐L2, LAG3, CTLA4, and TIM3 (Figure S1A). NUP107 was correlated (*r* > 0.3) with PD‐L1 (Figure S1C) and TIM3 (Figure S1D), and the correlation matrix is shown in Figure S1B. To summarize, NUP107 may promote HCC growth by dampening the immune response.

## 4 DISCUSSION

NUP107 is an important component of the core scaffold of the NUP160/NUP107 complex, which plays a major role in NPC assembly and cell cycle regulation.^32^ Several studies have demonstrated the overexpression of NUP107 in cervical cancer,^19^ colon cancer, lung cancer, and other tumors, although little is known regarding its role in liver cancer. In the present study, we analyzed NUP107 expression levels and its prognostic relevance in several HCC datasets, and explored the underlying mechanisms using bioinformatics tools. NUP107 was significantly upregulated in most human tumor tissues, including HCC, compared to the corresponding normal tissues, and correlated to more advanced HCC staging and worse prognosis. In addition, NUP107 expression had satisfactory diagnostic efficiency and predicted 1‐, 3‐, and 5‐year survival of HCC patients with high accuracy. The better ROC curves of NUP107 compared to some potential prognostic markers for HCC, such as CDK4^51^ and HMGA1,^52^ again demonstrate the satisfactory diagnostic value of NUP107. Thus, NUP107 is a prognostic and diagnostic biomarker for HCC.

Tumor cells differ from normal cells in terms of excessive proliferation, abnormal differentiation, and impaired apoptosis.^53^ Therefore, we also explored the molecular mechanisms underlying the role of NUP107 in HCC.^54^ GO and KEGG analyses indicated that NUP107 is enriched in cell cycle‐related pathways, and the results of GSEA also revealed significant association with the cell cycle, DNA replication, G2M checkpoint, E2F target, and mitotic spindle. The G2/M checkpoint prevents DNA‐damaged cells from entering the mitotic (M) phase^55^ and is regulated by the RB‐E2F complex that determines the timing and accuracy of cell cycle replication.^56^ NUP107 was closely associated with cell cycle regulatory genes such as BUB1B and MCM2, suggesting that NUP107 may regulate the cell cycle in HCC.

Targeted therapies^57^ and immunotherapy^58^ have significantly improved the outcomes in HCC patients. Therefore, the exploration of potential therapeutic targets and immune‐related molecules for HCC is crucial for its treatment. Tumor initiation, growth, progression, and metastasis are closely related to the tumor microenvironment,^59^ which also includes stromal cells like fibroblasts and infiltrating immune cells such as macrophages.^60^ The immune response to tumor cells can be a double‐edged sword, which can activate anti‐tumor pathways but also create an immunosuppressive microenvironment.^61^ Studies have shown that tumor infiltrating lymphocytes (TILs) can predict anterior lymph node status and survival in cancer patients.^62^ We observed higher infiltration of CD8+ T cells, cytotoxic T cells, DCs, mast cells, neutrophils, NK cells, pDCs, and γδ T cells in the NUP107^low^ group, and that of NK CD56^bright^ cells, NK cells, T helper cells, central memory T cells, follicular helper T cells, and Th2 cells in the NUP107^high^ group. Thus, NUP107 may influence the progression of HCC by regulating the tumor immune microenvironment.

Chemokines control the migration and recruitment of immune cells.^63^, ^64^ We found that NUP107 was positively correlated with chemokines such as CCL28 and CXCL8 (*r* = 0.310, *p* < 0.001), and the chemokine receptors CCR8 and CXCR4 suggesting that NUP107 overexpression may recruit immune cells to the tumor tissues. Activated CCL28 binds to CCR3 and CCR10, and can control the targeted migration of TILs, Tregs, and cancer‐associated stellate cells.^65^ Furthermore, CCR8 recruits TAMs and Tregs, and promotes tumor angiogenesis.^66^ The CXCR4‐CXCL12 axis attracts Tregs and pDCs to enhance tumor growth, which may be one of the mechanisms through which NUP107 promotes HCC growth.^67^

Targeted therapies against immune checkpoints, including programmed cell death‐1 (PD‐1)/programmed apoptosis ligand 1 (PD‐L1) and T‐cell immunoglobulin and mucin structural domain molecule 3 (Tim‐3), have been effective against solid tumors.^68^ The Tim‐3 inhibitor cabolimab and the PD‐L1 inhibitor atezumab are promising options for HCC patients.^69^, ^70^ The expression of immune checkpoints in tumor tissues regulates the degree of immune cell infiltration and also determines the response to immunotherapy.^71^ In the present study, we showed that NUP107 is positively correlated with PD‐L1 and Tim‐3, which suggests that the therapeutic effect of ICIs in HCC patients may be enhanced by targeting NUP107.

To summarize, we found that NUP107 is upregulated in HCC and portends poor prognosis, and can predict the survival of HCC patients with reasonable accuracy. NUP107 may exert its oncogenic effects on HCC via the regulation of cell cycle and immune infiltration. Our study has some limitations that ought to be considered. Firstly, the function of NUP107 in HCC cells needs to be validated by in vitro assays. Secondly, the mechanism by which NUP107 controls immune infiltration needs to be elucidated by animal experiments.

## 5 CONCLUSION

NUP107 correlated with the OS, RFS, and HCC staging in HCC patients and showed diagnostic accuracy. In addition, NUP107 was associated with cell cycle pathways, immune cell infiltration, and immune checkpoints. Thus, NUP107 is a reliable diagnostic and prognostic biomarker for HCC, as well as a potential therapeutic target. Our findings also provide new insights into the mechanisms associated with immune cell infiltration in HCC.

## AUTHOR CONTRIBUTIONS

**Ju‐sen Nong:** Writing – original draft (equal). **Xin Zhou:** Writing – original draft (equal). **Jun‐qi Liu:** Data curation (supporting). **Jian‐zhu Luo:** Software (supporting). **Jia‐mi Huang:** Software (supporting). **Hai‐xiang Xie:** Validation (supporting). **Ke‐jian Yang:** Validation (supporting). **Jing Wang:** Resources (supporting). **Xinping Ye:** Resources (supporting). **Tao Peng:** Supervision (lead).

## FUNDING INFORMATION

Key R&D Plan of Qingxiu District, Nanning (No. 2020056); Innovation Project of Guangxi Graduate Education (YCSM2022227, 02603222064X).

## CONFLICT OF INTEREST STATEMENT

The authors have declared that there are no competing interests.

## ETHICS STATEMENT

This study had acquired the approval of the Ethics Committee of the first affiliated hospital of Guangxi Medical University before specimen collection. Approval Number: 2022‐KY‐E‐159. Written informed consent was provided by each patient.

## Supporting information


Figure S1.

Figure S2.

## Data Availability

The datasets used and/or analyzed during the current study are available from the corresponding author on reasonable request.
