# Supplementary figures and images for: Nucleoporin 107 is a prognostic biomarker in hepatocellular carcinoma associated with immune infiltration
Source: Cancer Med. 2023 Mar 23;12(9):10990–1009. doi: 10.1002/cam4.5807 (PMC10225238; doi:10.1002/cam4.5807)

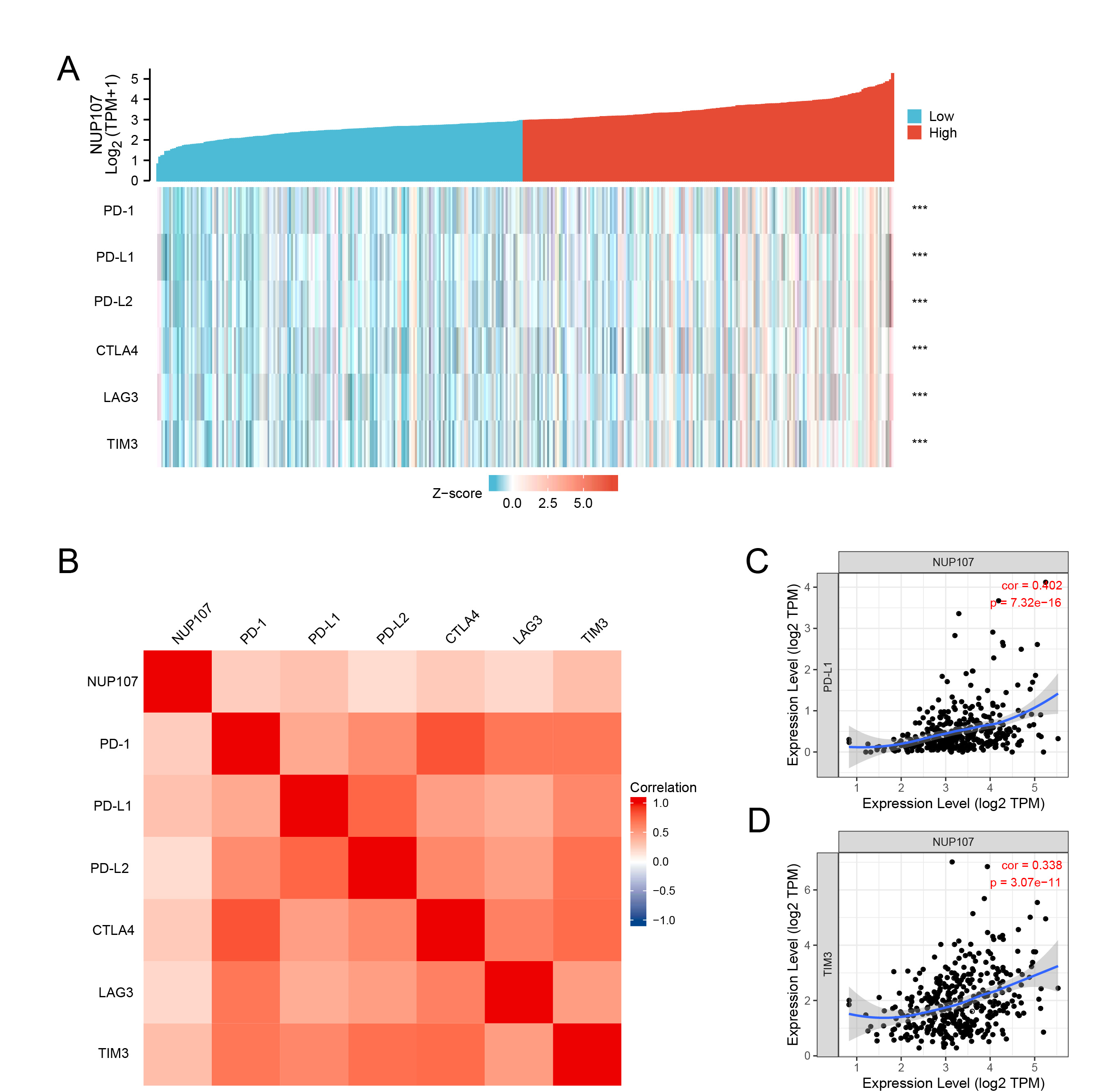

Supplement: Supplementary file 1 — Figure S1. [file CAM4-12-10990-s001.jpg]

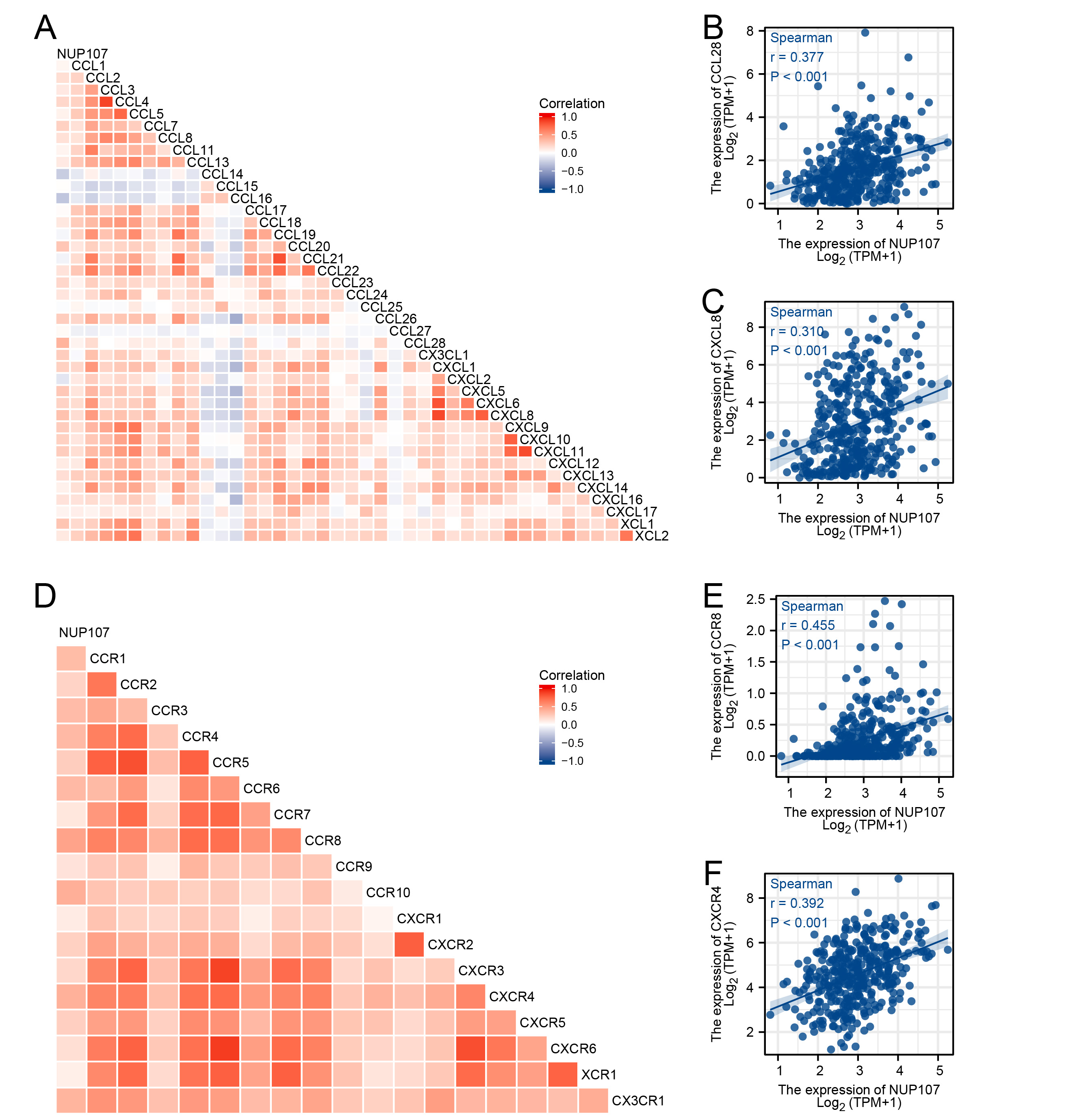

Supplement: Supplementary file 2 — Figure S2. [file CAM4-12-10990-s002.jpg]
